# 

**Published:** 1860-12

**Authors:** 


### CONTENTS.

ORIGINAL ESSAYS AND COMMUNICATIONS.

The Province of the Dentist. By J. Taft, ..... 313
Treating and Capping Exposed Nerves. By Dr. W. Pease, ..... 316
Lord Oxygen. By Geo. Watt, ..... 321
The Anatomy. Physiology, Pathology, and Remedial Treatment of the Fifth Pair of Nerves. By J. H. M'Quillen, D. D. S. ..... 330
Removal of the Lower Jaw, for Osteo-Sarcoma of Immense size By Geo. C. Blackman, 341
Patent Instruments. By J. A. Murphy, M. D. ..... 350

PROCEEDINGS OF SOCIETIES.

Minutes of the Cincinnati Local Dental Association ..... 358
Editorial ..... 362
Business Notices ..... 368

Communications must reach the Editor as early as the 15th, to insure insertion in the next issue.

